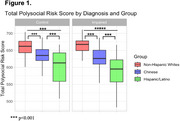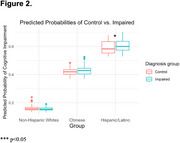# Social Determinants of Health and Cognitive Impairment:The Role of the Polysocial Risk Score

**DOI:** 10.1002/alz70860_107392

**Published:** 2025-12-23

**Authors:** Stefanie D. Pina‐Escudero, Jorge Archila‐Puac, Oscar Robles Archila, Gloria A. Aguirre, Valentina E. Diaz, Karen A. Dorsman, Kevin Lieu, Diana Mei, Andjelika Milicic, Ashley J. Jackson, Berenice Fuentes‐Juarez, Maritza Pintado‐Caipa, Jennifer S. Yokoyama, Julio C. Rojas, Gil D. Rabinovici, Joel H Kramer, Katherine L. Possin, Charles C. Windon, Boon Lead Tee, Serggio Lanata

**Affiliations:** ^1^ Memory and Aging Center, Weill Institute for Neurosciences, University of California San Francisco, San Francisco, CA, USA; ^2^ Global Brain Health Institute, University of California, San Francisco, San Francisco, CA, USA; ^3^ Global Brain Health Institute, University of California, San Francisco, CA, USA, San Francisco, CA, USA, San Francisco, CA, USA; ^4^ UT Southwestern Medical Center, Dallas, TX, USA; ^5^ Memory and Aging Center, UCSF Weill Institute forNeurosciences, University of California, San Francisco, San Francisco, CA, USA, San Francisco, CA, USA; ^6^ Memory and Aging Center, UCSF Weill Institute for Neurosciences, University of California, San Francisco, San Francisco, CA, USA

## Abstract

**Background:**

The Polysocial Risk Score (PSRS) is derived from the UCSF Memory and Aging Center (MAC) Social Determinants of Health (SDOH) questionnaire, which evaluates lifelong SDOH factors across various domains, including parental background, education, employment, economic stability, healthcare access, housing stability, nutrition, and experiences of adversity and discrimination. The questionnaire is administered to research participants at the MAC in English, Chinese (Traditional or Simplified), and Spanish, with proxies assisting cognitively impaired individuals when necessary. Due to the availability of the questionnaire in multiple languages participants typically self‐identify with one of the following groups: non‐Hispanic whites, Chinese, or Hispanics/Latinos.

**Methods:**

An unweighted cumulative PSRS was calculated by summing all questionnaire variables, with scores ranging from 0 (maximum adversity) to 764 (minimal adversity). Non‐parametric tests and logistic regression models were used to evaluate the association between PSRS and cognitive impairment, defined as a diagnosis other than “control” by a multidisciplinary team.

**Results:**

A total of 506 participants were evaluated, with 33.2% male and a mean age of 72.74 ± 10.35 years. Of these, 33.5% were cognitively impaired. The prevalence of cognitive impairment was highest among Hispanics/Latinos (65.7%), and lowest among non‐Hispanic whites (19.9%). The mean PSRS across the cohort was 498.37 ± 160.70, with the highest mean observed in the non‐Hispanic white group (517 ± 169). After grouping participants according to cognitive status, PSRS differed significantly between groups in both controls (*p* < 0.001) and cognitively impaired individuals (*p* < 0.001) (Figure 1). However, PSRS was only predictive of cognitive impairment in the Hispanic/Latino group (*p* = 0.04) (Figure 2).

**Conclusion:**

Lower PSRS scores, indicating greater adversity, were observed in Hispanic/Latino participants and were significantly associated with cognitive impairment in this group. A larger sample size and a weighted scoring approach will be necessary to further explore the contribution of SDOH to cognitive impairment in these populations.